# Depression severity mediates stigma and quality of life in clinically stable people with schizophrenia in rural China

**DOI:** 10.1186/s12888-023-05355-x

**Published:** 2023-11-11

**Authors:** Siyuan Zheng, Ruoqi Wang, Shaofei Zhang, Yangxu Ou, Xuanlian Sheng, Meng Yang, Menglin Ge, Lei Xia, Jun Li, Xiaoqin Zhou

**Affiliations:** 1 School of Mental Health and Psychological Sciences, Anhui Medical University, Hefei City, China; 2 Department of Psychiatry, Chaohu Hospital of Anhui Medical University, Hefei City, China; 3 Anhui Psychiatric Center, Hefei City, China; 4 Department of Psychology and Sleep Medicine, The Second Affiliated Hospital of Anhui Medical University, Hefei City, China

**Keywords:** Schizophrenia, Stigma, Depressive symptoms, Quality of life, Influencing factors

## Abstract

**Background:**

Depressive symptoms associated with schizophrenia are closely related to stigma and quality of life(QOL). There is, however, no thorough research on the connection between the three. This study sought to investigate the possible factors influencing depressive symptoms in people with schizophrenia (PWS) in rural Chaohu, China, and to further explore the role of depression severity in stigma and lifestyle quality.

**Methods:**

Eight hundred twenty-one schizophrenia patients accomplished the entire scale, including the 9-item Patient Health Questionnaire (PHQ-9), the Social Impact Scale (SIS), and the World Health Organization on Quality of Life Brief Scale(WHOQOL—BREF). A straightforward mediation model was employed to determine if the intensity of the depression could act as a mediator between stigma and QOL.

**Results:**

Two hundred seventy-nine schizophrenia patients (34%) had depressive symptoms (PHQ ≥ 10), and 542 patients (66%) did not (PHQ < 10). Logistic regression showed that marital status, job status, physical exercise, standard of living, and stigma contributed to the depressed symptoms of schizophrenia. Depression severity partially mediated the effect between stigma and QOL, with a mediating effect of 48.3%.

**Conclusions:**

This study discovered a significant incidence of depressed symptoms associated with schizophrenia, with depression severity serving as a mediator variable connecting stigma and QOL and partially moderating the association.

## Introduction

Anomalies in cognition, thought, emotion, and behaviour characterize a collection of extreme mental illnesses known as schizophrenia. It is one of the most debilitating disorders and can often cause extensive disability in daily functioning and cognition [1]. Many clinical studies in the past have focused on the pathological symptoms of schizophrenia [2]. However, with the improvement of patients' psychiatric symptoms, their daily life problems are gradually exposed, including employment, housing, social function, and social relations [3]. Therefore, to provide more comprehensive services for people with schizophrenia (PWS) and help them return to the community as soon as possible, an increasing number of studies are focusing on their quality of life(QOL) [4, 5].

QOL is defined by whether an individual can meet their essential needs and social obligations while utilizing their skills to exploit the opportunities provided by society [6]. QOL is an outcome indicator for evaluating the treatment of PWS [7, 8]. A previous meta-analysis of 18 case–control studies found that the QOL of PWS was significantly lower than that of healthy controls [9]. Additionally, it was discovered in another study that QOL can predict the return of symptoms in schizophrenia patients. According to the study, the likelihood of relapsing after two years of follow-up increases with a decreased QOL [10].

The QOL of PWS is affected by numerous factors, and cross-sectional and longitudinal studies have identified several determinants of QOL in patients with severe mental disorders, such as symptom severity, the presence of depression and anxiety, medication side effects, and treatment adherence [11, 12]. In PWS, lower QOL is associated with the presence of a secondary diagnosis, such as depression [13, 14] or anxiety [15]. PWS frequently experience depressive symptoms (7–65%).Those symptom can be seen in almost all onset stages [16] and are associated with the use of multiple medications, poor psychosocial functioning, low QOL, etc. [17, 18]. Numerous studies have revealed a link between depressive symptoms and poor QOL in PWS [19, 20]. Higher rates of recurrence and readmission have been reported in PWS with depressive symptoms than in non-depressed controls [21]. Furthermore, depression is also a prevalent warning indicator for suicidal thoughts and actions, and many prospective studies have found that depression poses a significant risk of suicidal ideation, conduct, and fatalities [22, 23]. However, because depressive symptoms and the adverse symptoms of schizophrenia frequently coexist, depressive symptoms in schizophrenia are frequently under-recognized and under-diagnosed, which prevents patients from receiving appropriate treatment [24].

Stigma is an internal experience of shame that patients experience as a result of their illness [25]. It can be divided into 'social stigma', which refers to the experience of discriminatory attitudes and unfair treatment by the public, and 'self-stigma', which alludes to the shame that prevents patients from sharing their experiences, asking for help from others, and experiencing the anticipated sentiments of discrimination [26, 27]. In a 2018 cross-sectional study survey, it was found that a significant proportion of PWS are stigmatized, with greater hopelessness, depression and suicidal risk all being linked to stigma [28].

First, many studies have shown that both internal and social stigma significantly affect the QOL of PWS [29, 30]. For example, it can lead to social exclusion, preventing patients from engaging in meaningful life activities [31, 32] and reducing their self-esteem and self-efficacy [33], which are important components of self-concept, and the deterioration of this self-concept may produce a number of negative outcomes that may affect their recovery outcomes. In addition, stigmatization can hinder early treatment and recovery of people with mental illness, leading to prolonged recovery time, which further reduces QOL [34–36]. Second, stigma can limit the ability of PWS to express their distress and seek help, thereby impairing their social interactions and increasing their risk of depression [37, 38]. The issue of stigma was also found in a longitudinal study to significantly affect PWS, who feared disclosure of their illness, and the results of that study suggested that the more stigmatized the patient was, the more depressed he or she felt [39]. Although the results of the association remain contradictory, according to a meta-analysis of these studies, stigma still has nearly a one-in-three chance of predicting depression [40].Third, many studies have shown that depressive symptoms in PWS can affect social function to varying degrees, reducing the QOL [41]. Yoshimune et al. studied the relationship between QOL and clinical factors in hospitalized schizophrenic patients and found that depressive symptoms significantly affected the QOL of the patients, whereas other symptoms such as positive, negative and pharmacogenetic extrapyramidal symptoms did not have a significant effect on their QOL [42]. Two other studies that included 174 and 67 outpatients with schizophrenia, respectively, reported a significant correlation between QOL and depressed mood but no significant correlation with positive and negative symptoms and extrapyramidal side effects [43, 44].

In summary, we can see that stigma can reduce the QOL of PWS and that stigma can lead to a greater likelihood of depression, and depressive symptoms can further reduce QOL. Therefore, we hypothesized that the severity of depression might be a potential factor for stigma to reduce the QOL of PWS; that is, the severity of depression plays a mediating role between stigma and QOL.

Although depressive symptoms and stigma associated with schizophrenia can affect the QOL, no prior research has studied the connection between the three in this group. The objectives of this study were 1) to assess the demographic and clinical correlates of depressive symptoms associated with schizophrenia; 2) to develop a mediation model to investigate how the severity of depression impacts stigma and living conditions.

## Methods

### Participants

PASS 15.0 was used to calculate the sample size for this study. Based on previous literature estimating that the probability of schizophrenic patients experiencing depressed mood is approximately 40% [45], with a specified tolerance error of 4% and a confidence level of 1-α = 0.95, the above software was used to calculate the sample size to be surveyed as *N* = 599; assuming a non-response rate of 20% among the study population, a sample size of *N* = 749 would be needed.

In this multicentre cross-sectional study, researchers visited 6 streets and 12 townships in the Chaohu area from September to October 2022; all participants were from the rural area of Chaohu City, Hefei City, Anhui Province, and all were enrolled in the database system for the management of severe mental disorders in Chaohu City, Anhui Province, which has more than 3,000 registered users. A total of 1205 questionnaires were sent out and 821 were validly returned, according to 3:1 random stratified sampling. The inclusion criteria were as follows: 1) diagnosis of schizophrenia according to ICD-10 review of medical records; 2) diagnosis as clinically stable by a psychiatrist, which according to previous studies [46, 47] can be defined as less than 50% change in dose of any primary psychotropic medication in the last three months; 3) age 18–75 years; 4) no other psychiatric disorders; 5) informed consent signed by the patient or her legal guardian. The exclusion criteria were as follows: 1) history of neurological disorders or substance abuse; 2) severe physical illness; 3) unwillingness or inability to participate in the assessment.

Our work received approval from the Chaohu Hospital Affiliated with Anhui Medical University Ethics Committee with the ethics number 202212-kyxm-13. All research procedures were strictly in line with the principles of the Helsinki Declaration. Additionally, all participants willingly completed informed consent forms, along with their legal representatives.

### Measures

#### General details and clinical characteristics

We utilized a broad questionnaire we created ourselves to collect all participants' age, gender, religion, marital status, grade (junior high school and below; high school/secondary; undergraduate/college), job status, average income, medication use, times of hospitalizations, age at onset, and duration of illness.

#### Depressive symptoms

We used the Patient Health Questionnaire (PHQ-9) to assess depressive symptoms in PWS. The PHQ-9 scale was compiled by Spitzer et al. [48], and was formulated according to the 9 symptoms of DSM-IV depressive disorder. It is widely used in scientific research and clinical practice because of its simplicity and easy operation. The total PHQ-9 score ranged from 0 to 27 points, and the standard cut-off value of possible major depression was 10 points or more [49, 50], established in the first study on the PHQ-9 [51]. In addition, Bian in China translated the scale into Chinese, which had good reliability and validity in applying it to general outpatients in public hospitals. The internal consistency coefficient was 0.857, and the sensitivity was 91% when the cut-off value was 10 [52]. Considering that when the cut-off value is 10, the specificity and sensitivity of the scale are higher, we defined < 10 as clinically free of depressive symptoms and ≥ 10 as clinically depressed. The current investigation used the scale as both a continuous and a categorical variable. The Cronbach’s alpha coefficient for the scale in this study was 0.818, with good internal consistency.

#### Stigma

To measure the severity of patient stigma, Fife et al. [53] created the Social Impact Scale (SIS), which Pan et al. [54] translated into Chinese in 2007. The scale contains 24 items in 4 dimensions: social rejection (9 questions), financial insecurity (3 questions), internalized shame (5 questions), and social isolation (7 questions). A 4-point Likert scale is used to grade each entry: 1 = strongly disagree, 2 = disagree, 3 = agree, and 4 = strongly agree. The total score for the scale is the sum of the 4-dimensional scores, with a total score from 24 to 96, with higher scores indicating more significant perceived stigma. The Cronbach’s alpha coefficient for this scale in this study was 0.927. The scale and its four dimensions were used in this investigation as continuous variables.

#### Quality of life(QOL)

We used the World Health Organization on Quality of Life Brief Scale ( WHOQOL-BREF) to assess the QOL of the subjects [55]. The scale was developed by the WHOQOL team in 1998 and is a reliable tool for evaluating QOL (Cronbach’s α = 0.78) [55, 56]. The scale consisted of a total of 26 items, of which the first two primarily examined the respondents’ overall subjective feelings about their QOL and health status. The other 24 items are divided into four categories: physiology, psychology, social interactions, and environment. According to how heavy an object was, it was given a score between 1 and 5 points. The initial scores for the four fields and subjective sensations were added to obtain WHOQOL-BREF’s overall score. The overall QOL improves as the score increases [57]. This study only used the first two separate items of the scale: 1)“How do you evaluate your quality of life ?”; 2) “How satisfied are you with your health ?”. However, previous studies have shown that these two individual items have good consistency with the other four fields [58, 59]. Therefore, we selected these two separate items to evaluate the QOL of the overall sample and used them as continuous variables in this study. The Cronbach's alpha coefficient for this scale in this study was 0.759.

### Statistical analysis

First, we compared schizophrenic patients with and without depressive symptoms. Independent samples t-tests or Mann–Whitney U-tests were used for continuous variables, and chi-square tests were used for categorical variables. Second, we examined variables affecting QOL and depressive symptoms in PWS using linear regression and forward logistic regression. In addition to general demographic characteristics, the entire stigma score and its four dimensions were included in the analyses. Finally, a bootstrap sample size of 5000 in Hayes' (2018) PROCESS SPSS (Model 4) was used to determine the mediating effect of depression severity on the stigma-QOL relationship.

## Results

### Demographic and clinical characteristics

Table 1 shows that 279 PWS were depressed, with a mean age of 50.64 ± 12.48 years and a greater number of females than males. Most of the participants had no religious beliefs and their education level was junior high school or below. Most of the participants were married, and most of the patients were unemployed, had low per capita family income, and were on psychiatric medication year-round. The majority of the patients had bad lifestyle habits, such as smoking and drinking. There were no significant differences between the two groups in terms of religion, education, duration of illness, medication use, or age of onset. In addition, there were significant differences between the two groups in terms of age, gender, marital status, work status, physical activity, average income, smoking, drinking, QOL, and total stigma scores (all *P* < 0.05), and the mean QOL scores were lower in patients with depressive symptoms than in those in the group without depressive symptoms (5.31 ± 1.15 vs. 6.33 ± 1.22, *P* < 0.05). The mean score of the SIS scale was higher in the group with depressive symptoms than in the other group (65.18 ± 9.20 vs. 58.82 ± 9.26, *P* < 0.05). In addition, some of the participants in this study had difficulty in recalling the duration of illness(*n* = 803), times of hospitalization (*n* = 795), and age at onset of illness (*n* = 804).

**Table 1:** Demographic and clinical characteristics in the total sample

Variables	Total samples (*n* = 821)	Group without depression (*n* = 542, 66%)	Group with depression (*n* = 279, 34%)	X^2^/Z	*P*
		(n%; mean ± SD)	(n%; mean ± SD)		
Age	821	542(48.26 ± 11.62)	279(50.64 ± 12.48)	-2.926	**0.003**
Gender					
Female	427	265(62.1)	162(37.9)	6.207	**0.013**
Male	394	277(70.3)	117(29.7)		
Religion					
No	701	464(66.2)	237(33.8)	0.065	0.799
Yes	120	78(65)	42(35)		
Educational level					
≤ Middle school	694	458(66.0)	236(34.0)	2.071	0.355
High school/Secondary school	100	63(63.0)	37(37.0)		
> High school	27	21(77.8)	6(22.2)		
Marital status					
No	378	272(72.0)	106(28.0)	11.020	**0.001**
Yes	443	270(60.9)	173(39.1)		
Job status					
No	668	405(60.6)	263(39.4)	46.389	**0.000**
Yes	153	137(89.5)	16(10.5)		
Physical exercise					
No	535	321(60.0)	214(40.0)	24.785	**0.000**
Yes	286	221(77.3)	65(22.7)		
Medication					
No	80	51(63.7)	29(36.3)	0.203	0.652
Yes	741	491(66.3)	250(33.7)		
Average income					
< 2000	739	477(64.5)	262(35.5)	7.130	**0.008**
≥ 2000	82	65(79.3)	17(20.7)		
Smoking					
No	671	428(63.8)	243(36.2)	8.153	**0.004**
Yes	150	114(76.0)	36(24.0)		
Drinking					
No	766	499(65.1)	267(34.9)	3.888	**0.049**
Yes	55	43(78.2)	12(21.8)		
Duration of illness	803	530(21.25 ± 11.15)	273(22.86 ± 12.30)	-1.480	0.139
Times of hospitalization	795	523(2.97 ± 3.86)	272(3.10 ± 3.70)	-0.739	0.460
Age at onset of illness	804	531(27.04 ± 11.13)	272(27.84 ± 13.32)	-0.185	0.853
WHOQOL-BREF score	821	542(6.33 ± 1.22)	279(5.31 ± 1.15)	-10.766	**0.000**
SIS total score	821	542(58.82 ± 9.26)	279(65.18 ± 9.20)	-9.349	**0.000**

Bolded *P* < 0.05

### Risk factors for QOL in schizophrenia

Table 2 lists the factors associated with QOL in PWS. A linear regression model was used to incorporate factors that may affect QOL into the independent variables. Model 1, which included the total SIS score as a dependent variable, found that patients' QOL was associated with average family income (OR = 0.167, 95% CI = 0.035–0.300, *P* < 0.05), total depression score(OR = -0.083, 95% CI = -0.098-(-0.068), *P* < 0.001), and total SIS score(OR = -0.021, 95% CI = -0.030-(-0.012), *P* < 0.001) and that patients' lower average family income, more severe depressive symptoms, and greater feelings of stigma led to a further decline in their QOL. The four dimensions of the SIS were included as independent variables in Model 2, and it was found that, in addition to average family income(OR = 0.173, 95% CI = 0.040–0.306, *P* < 0.05) and depressive mood(OR = -0.081, 95% CI = -0.096-(-0.066), *P* < 0.001), the more severe the social isolation (OR = -0.051, 95% CI = -0.092-(-0.010), *P* < 0.05) in the SIS scale, the worse the QOL of patients with schizophrenia.

**Table 2:** Logistic regression analysis on QOL

Variables	Model1		Model2	
	OR 95%CI		OR 95%CI	
Age	0.006	[-0.030,0.043]	0.007	[-0.029,0.043]
Gender(female = 0)	-0.004	[-0.205,0.198]	-0.003	[-0.204,0.198]
Religion(No = 0)	0.143	[-0.084,0.369]	0.153	[-0.073,0.379]
Educational level (≤ Middle school = 0)	-0.104	[-0.278,0.070]	-0.099	[-0.270,0.077]
Job status (No = 0)	0.153	[-0.167,0.374]	0.146	[-0.074,0.365]
Exercise status (No = 0)	-0.004	[-0.175,0.167]	-0.011	[-0.183,0.161]
Marital status (No = 0)	0.033	[-0.163,0.230]	0.027	[0.788, -0.170]
Average family income (< 2000 = 0)	0.167	[0.035, 0.300] *	0.173	[0.040,0.306] *
Smoking status (No = 0)	-0.070	[-0.300,0.160]	-0.075	[0.523, -0.306]
Drinking status (No = 0)	0.214	[-0.117,0.545]	0.208	[0.216, -0.122]
Age at onset of illness	-0.005	[-0.040,0.031]	-0.005	[-0.041,0.031]
Duration of illness	-0.001	[-0.037,0.034]	-0.002	[-0.037,0.034]
Times of hospitalization	0.013	[-0.008,0.034]	0.013	[-0.008,0.034]
Medication	0.141	[-0.145,0.427]	0.127	[-0.160,0.414]
Depression score	-0.083	[-0.098, -0.068] ***	-0.081	[-0.096, -0.066] ***
SIS total score	-0.021	[-0.030, -0.012] ***		
Social Rejection			-0.014	[-0.047,0.019]
Financial Insecurity			-0.037	[-0.105,0.030]
Internalized Shame			0.019	[-0.016,0.054]
Social Isolation			-0.051	[-0.092, -0.010] *
Constant	7.300		7.286	

*OR* odds ratio, *CI* confidence interval

^*^p < .05

^**^p < .01

^***^p < .001

### Depression risk factors in PWS

Table 3 lists the elements linked to depression symptoms in PWS. Factors associated with depressive symptoms in Table 1 were put into covariates to control for them, and conditional forward stepwise regressions were chosen. Controlling for confounders (including age, gender, smoking, alcohol consumption, and average income) revealed a strong relationship between patients' depressive symptoms and their marital status(OR = 0.552,95% CI = 0.392–0.777, *P* < 0.01), job status(OR = 4.486,95% CI = 2.478–8.122, *P* < 0.001), physical exercise (OR = 2.126,95% CI = 1.463–3.089, *P* < 0.001), quality of life (OR = 0.568,95% CI = 0.490–0.659, *P* < 0.001) and two dimensions of social rejection (OR = 1.078,95% CI = 1.009–1.151, *P* < 0.05) and social isolation (OR = 1.123,95% CI = 1.033–1.222, *P* < 0.05) on the SIS scale. According to these findings, depressive symptoms were more common in PWS who had a partner, no work, no physical exercise, a lower QOL, and more stigma.

**Table 3:** Logistic regression analysis on depression

Variables	Model1		Model2	
	OR	95%CI	OR	95%CI
Marital status(Yes = 0)	0.549	0.391–0.772**	0.552	0.392–0.777**
Job status(Yes = 0)	4.623	2.549–8.385***	4.486	2.478–8.122***
Physical exercise(Yes = 0)	2.187	1.507–3.172***	2.126	1.463–3.089***
WHOQOL—BREF score	0.561	0.484–0.651***	0.568	0.490–0.659***
SIS total score	1.063	1.043–1.084***		
Social rejection			1.078	1.009–1.151*
Social isolation			1.123	1.033–1.222*
Constant	0.066		0.064	

*OR* odds ratio, *CI* confidence interval

^*^p < .05

^**^p < .01

^***^p < .001

### Bivariate correlation

Table 4 shows the correlation analysis between the total SIS score of PWS patients and its four dimensions. There were significant correlations between the total SIS score and its four dimensions (*r* = 0.913, *P* < 0.01; *r* = 0.766, *P* < 0.01; *r* = 0.702, *P* < 0.01; *r* = 0.702, *P* < 0.01; *r* = 0.879, *P* < 0.01), and there were also a significant correlations among the four dimensions.

**Table 4:** Correlation between the total SIS score and its four dimensions

Variables	1	2	3	4	5
1.SIS total score	1				
2.Social Rejection	0.913**	1			
3.Financial Insecurity	0.766**	0.676**	1		
4.Internalized Shame	0.702**	0.508**	0.386**	1	
5.Social Isolation	0.879**	0.744**	0.641**	0.488**	1

^**^:*P* < 0.01

Table 5 displays the findings of the bivariate connection between total stigma scores, QOL, and total depression scores in PWS. There was a strong positive correlations between stigma and depression severity were found (*r* = 0.358, *P* < 0.001), as well as a significant negative correlations between quality of life and depression severity (*r* = -0.455, *P* < 0.001). In addition, there was a significant negative correlation between stigma and QOL (*r* = -0.318, *P* < 0.001) was also found. As a result of these findings, which showed a connection between depression severity, QOL, and stigma, depression severity (a continuous variable) was utilized as a mediating element in subsequent studies.

**Table 5:** Relationships between depression severity, stigmatization, and quality of life

Variables	1	2
1.PHQ-9 total score	1	
2.SIS total score	0.358***	
3.WHOQOL—BREF score	-0.455***	-0.318***

^***^:*P* < 0.001

### Depression severity as a mediator between stigma and QOL

**Fig. 1:**
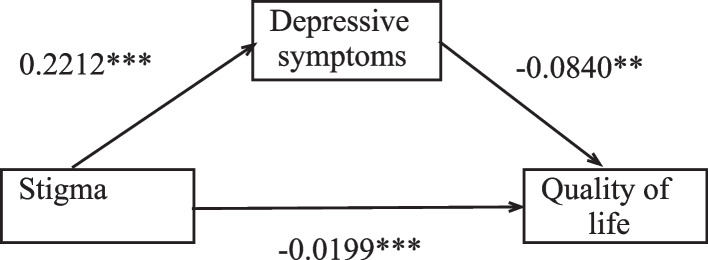
Mediation analysis showed an association between stigma, depression severity and QOL

According to the hypothesis proposed in this study, we use SPSS PROCESS (3.4) to establish a simple mediating model (Fig. 1). The model's independent, mediating, and dependent variables are stigma, depression severity, and QOL. The model was used to evaluate whether the severity of depression would mediate the impact of stigma on patients' QOL. The results of the mediation model are shown in Table 6. Stigma had direct [ β = -0.0199, *P* < 0.001] and indirect [ β = -0.0186, *P* < 0.001] effects on the quality of life of PWS, and the sum of the two was the total effect [ β = -0.0385, *P* < 0.001]. Bivariate correlation analysis between covariates (age, gender, marriage, work, physical exercise, income, etc.) was evaluated by the Mann–Whitney U-test or chi-square test (Table 1). After controlling the covariates, the statistical results showed that the 95% confidence interval of the indirect effect did not contain zero [ -0.0232, -0.0144], which indicated that the total depression score had a significant indirect effect on the relationship between stigma and QOL, and the mediating effect accounted for 48.3% of the impact of stigma on patients' living standards.

**Table 6:** Depression severity as a mediator between Stigmatization and QOL among study participants

	Total effect, *p*-value	Direct effect, *p*-value	Indirect effect, bootstrap confidence interval	Proportion mediated %
Quality of life(WHOQOL-BREF score)	-0.0385 ***P* < 0.001**	-0.0199 ***P* < 0.001**	-0.0186 [-0.0232, -0.0144]	48.3%

## Discussion

To our knowledge, this research is the first to examine how stigma and QOL in rural China are impacted by depressive symptoms associated with schizophrenia. The results showed that PWS with depressive symptoms had greater stigma levels and worse QOL, with the intensity of depression acting as a significant mediating factor between stigma and QOL.

This study indicated that approximately 34% of stable schizophrenia patients had depressive symptoms, which is consistent with other research on depression in schizophrenia patients in various sociocultural situations [60]. Contrary to earlier findings, our analysis revealed that PWS with a spouse were more likely to have depressive symptoms than those without a partner. Many studies have shown that marriage benefits physical and mental health, while divorce can be health-threatening [61]. However, while some people may experience long-lasting "scars" after separation or divorce, most people can quickly regain their health and happiness, and unhappy marriages might put couples in danger of poor intimacy and overall well-being [62].

Consistent with the findings of many studies, PWS who never engaged in physical activity were more likely than those who did to experience depressive symptoms [63, 64]. It has also been documented that appropriate physical activity not only reduces depressive symptoms in patients but also prevents cardiovascular disease [65]. The results of this study also show that PWS who are employed are less likely to experience depression than those who are not. Previous studies have well documented that employment provides productive benefits to patients and improves individual health and lifestyle quality [66, 67].

In recent studies, it was shown that the stronger the intensity of social rejection and social isolation in PWS, the more likely patients were to experience depression. Social exclusion has been linked to depression, which can lead to emotions of sadness and loneliness [68, 69]. Like significant physical sickness and QOL impairment related to despair, social isolation is also thought to be connected with mental health issues, particularly depression [70, 71].In this light, it is crucial to give PWS a social dimension. Research shows that social contact interventions between groups can reduce stigma [72].

The study's key conclusion is that the degree of depression partially mediates the link between stigma and life satisfaction. This finding is important because it has implications for therapies designed to lessen stigma and enhance PWS' living standards. First, in this study, the patient's living standards were significantly negatively correlated with the severity of stigma. Because of the history and understanding of the nature of the disease, people tend to reject PWS, and an interesting phenomenon in psychology is that the public tends to give socially desirable answers to controversial questions to maintain harmony [73]. Previous research has shown that the stigma of mental illness is far greater in Chinese culture than in Western culture [74]. Deeply rooted and specific cultural and philosophical ideas can contribute to stigma and act as barriers to remission and recovery. Traditional cultural values such as Confucianism and Taoism, which have influenced society for more than 2,000 years, also include the issue of "face" [75]. In Chinese society, people are first and foremost seen as living in a strict network of social interactions, and the maintenance of society depends on human interaction. As "face" represents power and status in Chinese society, preserving "face" is part of people's daily lives, and a diagnosis of schizophrenia can lead to a "loss of face", causing the individual to suffer from extreme symptoms of schizophrenia. A diagnosis of schizophrenia can lead to a "loss of face" for the individual, resulting in an extreme sense of shame for the patient [76–79]. In addition, some Chinese people believe that having a mental illness in this life means that they committed a moral wrong in a previous life, so that people with mental disorders are perceived by the public as being completely uncultivated and incapable of participating in social activities [80]. For example, in a survey of suicides in rural China, PWS were not accounted for, suggesting that people with schizophrenia were not considered morally competent [81].

In a collectivist society, people are more likely to recognize the public's stigmatization of mental illness than to express their values. This phenomenon severely limits and reduces the social status of patients. Schizophrenic patients are often considered different from others and are therefore negatively labelled, making it challenging to integrate into the community [82, 83]. Previous research has also demonstrated that stigmatization can lead to patients imposing negative stereotypes about themselves, resulting in self-discrimination and isolation [84] and a gradual withdrawal from social activities such as learning, working, and socializing, ultimately lowering their QOL [85]. Second, in this study, stigma is positively correlated with the severity of depression, and stigma can also indirectly affect the living standards of patients through the degree of depression (Fig. 1), which means that depression acts as a 'bridge.' Studies have found that stigmatization can result in slower healing rates and psychological issues [86]. For example, a previous cross-sectional survey showed that discrimination could hurt the rehabilitation of people with schizophrenia spectrum disorders through depressive symptoms [87]. In addition, a longitudinal survey also confirmed the hypothesis that stigmatization is a factor leading to higher depressive symptoms [39]. Outpatients with prejudices about mental illness who do not want to believe that their symptoms will improve and thus behave in a more depressed manner [34, 36]. These aggravated depressive symptoms can reduce the patient's ability to work and result in their refusal to enter society, thus reducing their economic income and ultimately worsening their QOL [88]. Based on the above statements, our results may imply that one of the causes of the continued decline in the level of well-being for PWS who experience stigma is the intensity of depression.

Thus, these findings can guide mental health workers to develop effective measures for the rehabilitation of PWS. For example, due to the lack of public knowledge of mental illness, people tend to attribute the symptoms of the disease to patients, so it is essential to educate the public about the aetiology, symptoms, and treatment of schizophrenia [89]. In this era, when social media is widely used, we can educate the public through many platforms, so that patients can be understood, respected, and accepted. Assertive Community Treatment (ACT) can be used to provide multidisciplinary care for patients with mental illness. The team includes psychiatrists, nurses, and social workers who can assist patients with daily life needs such as disease treatment, drug management, work, housing, and transportation [90]. Finally, clinicians in the treatment of schizophrenia also need to pay attention to other interventions, such as cognitive behavioural therapy or social skills training, which may improve symptoms, dysfunctional attitudes, and functions to reduce self-stigma and depressive symptoms, thereby improving the patient's psychological well-being [91, 92].

The scope of this investigation has several limitations. First, as this is a cross-sectional study, it is not possible to elaborate on the causal relationship between variables but only to assess the correlation, so future longitudinal studies are needed for more in-depth exploration. Second, despite the extensive assessment of patient demographic and clinical characteristics, there is evidence that factors such as sleep, diet, and type of antipsychotic medication all influence depressive symptoms in PWS [93–95], and these factors could be examined in the future.

## Conclusion

According to studies, depressive symptoms can occur in up to 34% of PWS, and the intensity of depression may partially influence the link between stigma and life expectancy. To lessen the intensity of depression and further enhance patient QOL, health workers must pay close attention to the depressive symptoms of such patients, come to an agreement on the diagnosis of the illness, and develop efficient treatment and prevention measures. Additionally, more studies are required to validate the efficacy of antidepressant medication for PWS depression symptoms because there are currently few data [96].

## Data Availability

The datasets used and/or analyzed during the current investigation are accessible from the corresponding author upon justifiable request, and all data supporting our conclusions are included in the publication.

## Abbreviations

PWS: People with schizophrenia
QOL: Quality of life
PHQ-9: 9-Item Patient Health Questionnaire
SIS: The Social Impact Scale
WHOQOL – BREF: The World Health Organization on Quality of Life Brief Scale
